# Cone-Beam Computed Tomography Correlates with Conventional Helical Computed Tomography in Evaluation of Lipiodol Accumulation in HCC after Chemoembolization

**DOI:** 10.1371/journal.pone.0145546

**Published:** 2016-01-11

**Authors:** Toru Ishikawa, Satoshi Abe, Asami Hoshii, Yumi Yamada, Akihiko Iiduka, Takeo Nemoto, Keiko Takeda, Toshiaki Yoshida

**Affiliations:** 1 Department of Gastroenterology and Hepatology, Saiseikai Niigata Daini Hospital, Niigata, Japan; 2 Department of Medical Radiology, Medical Radiographer, Saiseikai Niigata Daini Hospital, Niigata, Japan; 3 Department of Radiology, Saiseikai Niigata Daini Hospital, Niigata, Japan; University of Modena & Reggio Emilia, ITALY

## Abstract

**Background & Aims:**

The amount of drug-loaded lipiodol in an HCC tumor post-transarterial chemoembolization (TACE) correlates with the risk of local tumor recurrence. Lipiodol enhancement of a tumor on conventional CT, measured in Hounsfield units (HU), can predict tumor response. Here we investigate whether cone-beam CT (CBCT) can also be used to predict tumor response, providing the benefit of being able to optimize the patient’s treatment plan intra-procedurally.

**Methods:**

A total of 82 HCC nodules (82 patients), ≤5 cm in diameter, were treated with balloon-occluded TACE using miriplatin between December 2013 and November 2014. For each patient, both CBCT and conventional CT images were obtained post-TACE. The degree of correlation between CBCT and conventional CT was determined by comparing identical regions of interest for each imaging modality using pixel values.

**Results:**

The pixel values from conventional CT and CBCT were highly correlated, with a Pearson correlation coefficient of 0.912 (p<0.001). The location of the nodules within the liver did not affect the results; the correlation coefficient was 0.891 (p<0.001) for the left lobe and 0.926 (p<0.001) for the right lobe. The mean pixel value for conventional CT was 439 ± 279 HU, and the mean pixel value for CBCT was 416 ± 311 HU.

**Conclusions:**

CBCT may be used as a substitute for conventional CT to quantitatively evaluate the amount of drug-loaded lipiodol within an HCC nodule and, hence, the efficacy of TACE treatment. The major benefit of using CBCT is the ability to predict the likelihood of local recurrence intra-procedurally, enabling subsequent treatment optimization.

## Introduction

Miriplatin (MPT; Dainippon Sumitomo Pharm, Co. Ltd, Osaka, Japan) is a new drug specifically designed for transarterial chemoembolization (TACE) of hepatocellular carcinomas (HCCs) in Japan [1, 2]. MPT administered with balloon-occluded TACE (B-TACE), a modification of conventional TACE believed to improve drug concentration in the tumor [3], was shown to be more effective than conventional TACE by improving patient survival [4, 5]. Though MPT was also shown to cause less arterial damage than the other TACE drugs epirubin and mitomycin C, Miyayama et al. recently reported that superselective TACE using MPT increased local recurrence rates [6].

We have previously reported that the average Hounsfield unit (HU) value of a tumor obtained from conventional CT after balloon-occluded TACE (B-TACE) with MPT is predictive of the recurrence risk [7]. The logistical challenges with this approach however, is that no intra-procedural treatment optimization can occur, nor can the patient be counseled on the subsequent treatment regimen until the conventional CT has been conducted.

Cone-beam CT (CBCT), an imaging methodology already well-established in the diagnosis of HCC [8], may present a valuable intra-procedural alternative to conventional CT in the evaluation of TACE procedures. Recently, Wang et al. compared the capability of assessing lipiodol (Lipiodol Ultra-Fluid; Dainippon Sumitomo Pharma Co., Ltd, Osaka, Japan) deposition between CBCT and conventional CT and reported a high correlation between the two. The study was conducted using a complicated technique with three dimensional software and only 31 target tumors [9]. Here we corroborate their results using a simplified technique in a larger patient cohort.

## Patients and Methods

### Study Cohort

The study group included patients who were treated with B-TACE using MPT between December 2013 and November 2014. All patients received a comprehensive evaluation by dynamic contrast CT and MRI prior to treatment. The study exclusion criteria were: 1) tumor size ≥ 5 cm; 2) tumor infiltration; 3) ≥ 4-month interval between TACE and initial CT during follow-up observation; 4) recurrent HCC; 5) prior HCC treatment; and 6) no CT done during follow-up observation.

### TACE Protocol

In all cases, vascular access was achieved with the Seldinger technique. Briefly, the femoral artery was punctured, and a 5-Fr introducer was inserted followed by a 5-Fr catheter. A micro-balloon catheter (Attendant, Terumo, Tokyo, Japan or Logos, Piolax, Kanagawa, Japan) was then advanced into selective or super-selective branches of the tumor’s feeding arteries through a 5-Fr catheter.

MPT was utilized according to the "Guideline on the Use of New Anticancer Drugs for the Treatment of Hepatocellular Carcinoma" [2]. Each 70-mg vial of MPT was dissolved in 4 mL of lipiodol to prepare, at most, an 8-mL suspension of lipiodol with 140 mg of MPT. MPT was injected into the occluded artery under the inflated balloon catheter until the HCC nodule was filled with MPT.

### Cone Beam CT Imaging

CBCT was conducted immediately post-embolization using an AlluraClarity FD20 (Philips, Best, The Netherlands). Six hundred projection images were obtained by 5.2-s acquisitions with 240°C-arm rotation around the patient using an X-ray tube voltage of 117–123 kV, a pulse width 5–10 msec, and a tube current 50–325 mA for 166 nGy/frame detector dose. The flat panel detector was used for image acquisition, which has a focal spot-detector distance of 120 cm with 30 × 38 cm effective image area. The images were automatically transferred to an Xtravision workstation (Philips) and 3D images were reconstructed for a 250 ×250 ×194 mm field of view (matrix size 256 × 256 ×198) with a voxel size of 0.98 mm^3^, and artifact reduction (white compression restoration, over-scan correction, scatter correction, gain correction, ring artifact correction). In preparation of the scan, EKG leads, blood pressure cuffs and saturation monitor cables were temporarily removed. Patients were instructed to hold their hands above their heads and hold their breath at end-expiration during the CBCT scanning.

### CT Imaging

All conventional CT images were obtained using a multi-detector-row helical CT scanner (Aquilion PRIM; Toshiba Medical Systems, Tokyo, Japan) and a standard abdominal helical scan protocol. The tube voltage was 120kVp; the rotational time was 0.5 seconds; detector collimation was 0.5 mm/row; and helical pitch factor was 1.388/revolution. The images were reconstructed for a 350 × 350 mm field of view (matrix size 512 × 512) with a voxel size of 0.68mm^3^.

### Tumor segmentation and lipiodol accumulation

**Fig 1:**
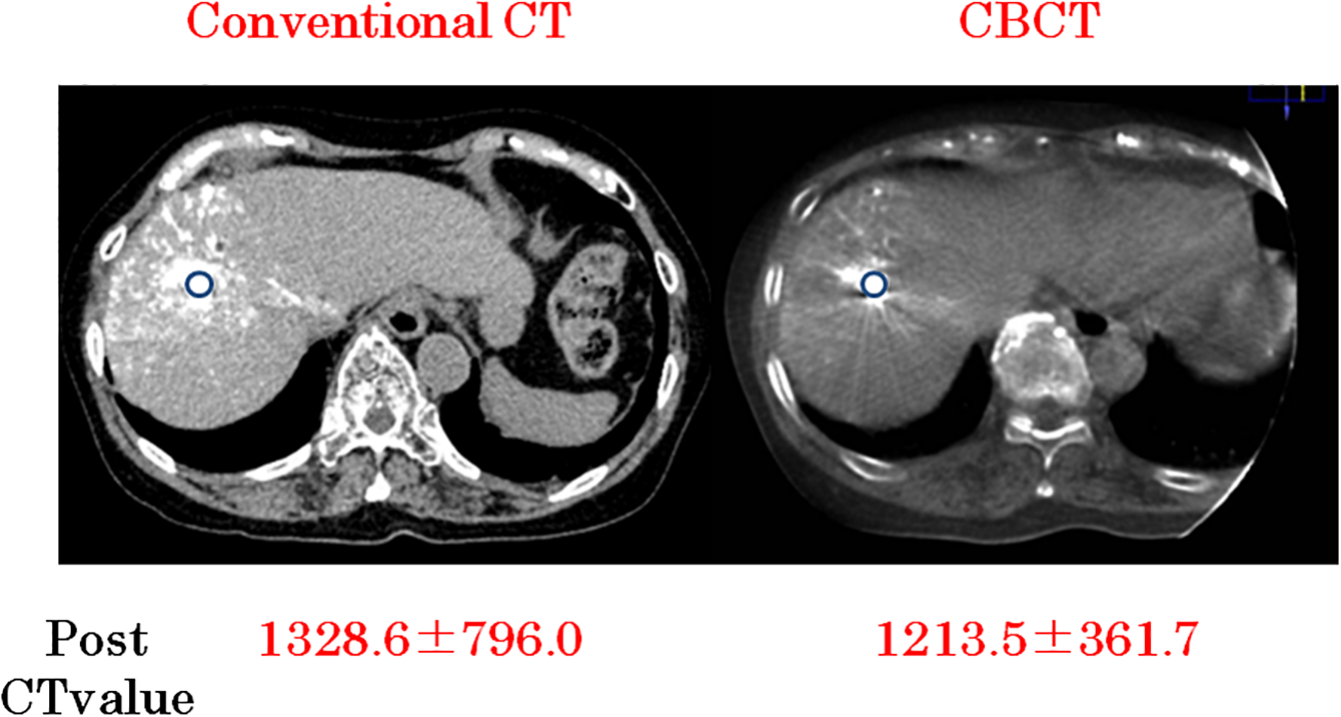
Comparison of ROI on conventional CT (scan parameter; 120kVp, 1366mAs, total scan time; 2.8 seconds, slice thickness; 0.98mm) and CBCT (scan parameter; 119kVp, 165mA, total sweep time; 5.2 seconds, slice thickness; 0.68mm)

Both CBCT and conventional CT images were transferred to the PACS as DICOM data. Using the EV Insite net software, regions of interest (ROIs) were placed on both CBCT and conventional CT DICOM images, adjusted to 1-mm slice thickness and situated in the same position (Fig 1). The automatically generated average HU value was recorded. Each scan was retrospectively evaluated by three hepatologists.

### Ethics Statement

Data are available from the Saiseikai Niigata Daini Hospital Data Access/ Ethics Committee for researchers who meet the criteria for access to confidential data.

The study was approved by the Institutional Review Board of Saiseikai Niigata Daini Hospital and was conducted in accordance with the principles of the Declaration of Helsinki. All patients provided written informed consent.

### Statistical Analysis

The correlation was analyzed using Pearson’s correlation coefficient, with P<0.05 considered significant.

## Results

The study included 82 patients (65 men and 17 women; mean age 71.4 ± 7.7 years). A total of 82 nodules were treated with balloon-occluded TACE. The average diameter on conventional CT was 31.3 ± 5.8 mm. The average pixel value for the ROI from conventional CT (plain CT value) and from CBCT (CBCT value) was 438.7 ±279.0 and 416.1 ±311.1, respectively (Table 1).

**Table 1:** Clinical background characteristics of 82 nodules.

Demographic variable	Mean ±SD	Range
Age (years)	71.4 ± 31.3	55–85
Sex (Male: Female)	65: 17	
Etiology (HBV/HCV/NonBNonC)	13/ 40/29	
Size (mm)	31.3 ± 5.8	20–45
Location(S1/S2/S3/S4/S5/S6/S7/S8)	0/7/9/13/6/15/6/26	
Conventional CT value(Hounsfield Units)	438.7 ± 279.1	145.5–1148.2
CB-CT value	416.1 ± 311.1	161.1–1443.9

**Fig 2:**
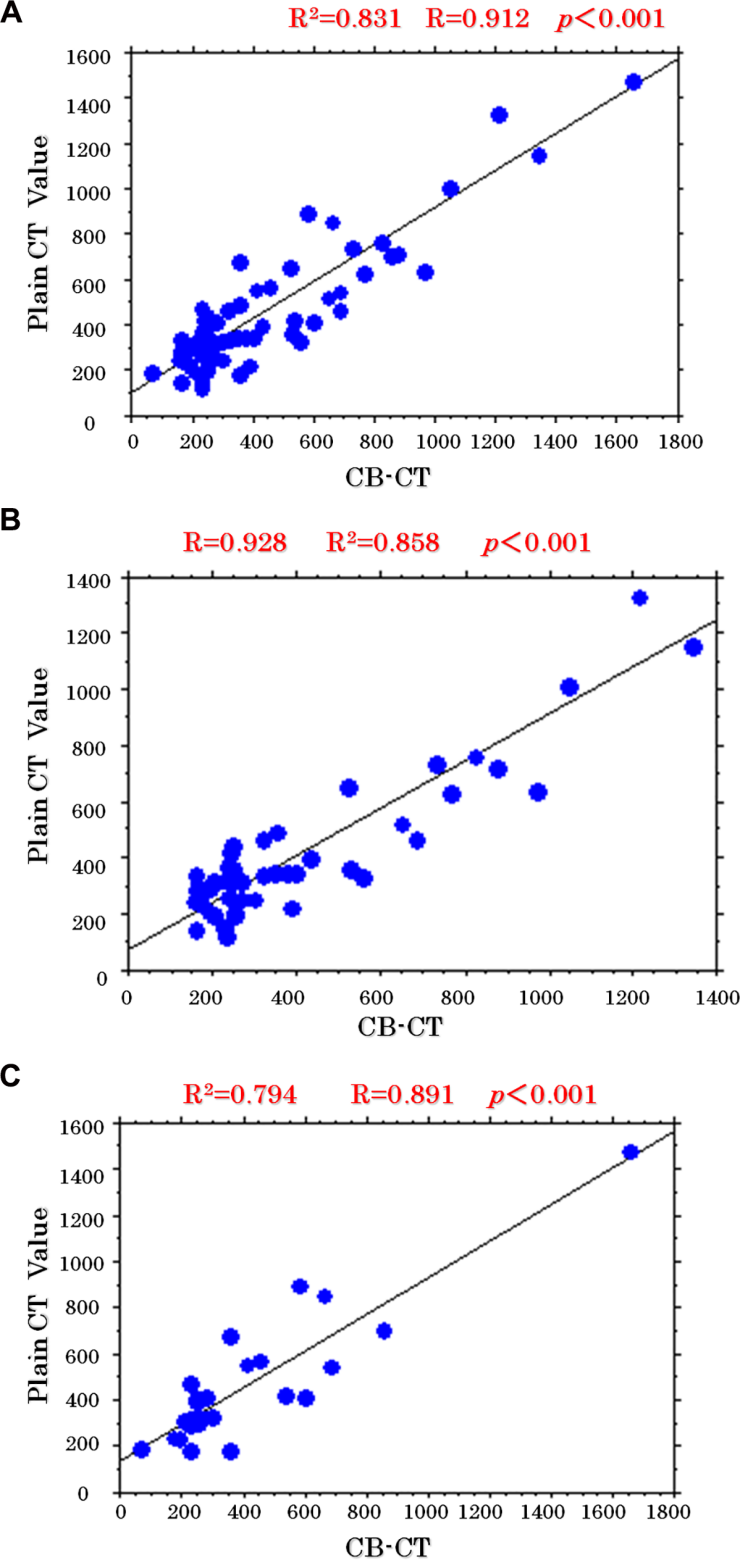
a) Correlation of CB-CT and conventional CT values. b) Correlation of CB-CT and conventional CT values. c) Correlation of CB-CT and conventional CT values.

There was a significant correlation between plain CT value and CBCT value, with a Pearson correlation coefficient of 0.912 (p<0.001) (Fig 2A). In the selected nodules of the left lobe of the liver, the correlation coefficient was 0.891 (p<0.001) (Fig 2B), and in the selected nodules of the right lobe of the liver, the correlation coefficient was 0.926(p<0.001) (Fig 2C).

## Discussion

Transarterial chemoembolization (TACE) is a treatment approach used when hepatocellular carcinoma (HCC) is not amenable to resection or ablation therapies. The effect of TACE depends on administering chemotherapy locally, rather than systemically, in addition to blocking the arterial supply to the tumor, thereby creating an ischemic core [10]. High concentrations of the drug-delivery vehicle lipiodol in the tumor at the time of treatment are associated with lower risks of local tumor recurrence [11]. Evaluating the effectiveness of TACE intra-procedurally enables physicians to optimize the treatment process for the patient. The high correlation between the pixel values from conventional CT and CBCT suggest that CBCT could be used to evaluate TACE efficacy.

The limitations of this approach were obtaining perfect alignment of the CBCT and CT images and specifying ROI location and size. To make a general standard of setting the ROI on the target lesion is also difficult, because the pixel value for the ROI is averaged even in a case having a partial defect of lipiodol accumulation. Nevertheless, the strength of the correlation and the agreement of the present findings with others [9] support the use of CBCT in the prediction of HCC tumor outcome post-TACE.

There was a negligible difference in the correlation between the left and right lobes that we attribute primarily to cardiac motion.

## Conclusion

A high correlation of pixel value for lipiodol accumulation in HCC tumors was shown between conventional CT and CBCT images. This provides a quantitative means of monitoring lipiodol accumulation, and enables the optimization of TACE treatment strategy both intra- and post-procedurally by predicting the risk of local tumor recurrence.
